# Assessing the impact of chemo-mechanical and soxhlet extraction techniques on cellulose nanofiber characteristics

**DOI:** 10.1371/journal.pone.0337323

**Published:** 2025-12-05

**Authors:** Muhammad Usman Zahid, Hifsa Zahid, Muhammad Usama Asghar, Waqas Khan Kayani, Aamir Rasheed, Faroha Liaqat, Faraz Muneer, Faiza Rasheed

**Affiliations:** 1 Department of Biotechnology, Faculty of Biological Sciences, Quaid-i-Azam University, Islamabad, Pakistan; 2 Department of Biotechnology, Faculty of Basic and Applied Sciences, University of Kotli, Kotli, Azad Jammu and Kashmir, Pakistan; 3 Department of Chemistry, University of Kotli, Kotli, Azad Jammu and Kashmir, Pakistan; 4 Department of Chemistry, Quaid-i-Azam University, Islamabad, Pakistan; 5 Department of Plant Breeding, Swedish University of Agricultural Sciences, P.O. Box 190, Lomma, SE-234 22, Sweden; National Research Centre, EGYPT

## Abstract

The extraction of cellulose nanofibers (CNFs) from lignocellulosic biomass provides a sustainable alternative to synthetic materials due to their biodegradability, mechanical strength, and environmental compatibility. However, conventional extraction methods are often affected by high chemical consumption, energy intensity, and limited scalability. This study presents a comparative and optimized approach for the sustainable extraction of CNFs using two distinct methods, including chemo-mechanical treatment and Soxhlet extraction, applied to sugarcane bagasse and eucalyptus bark. Unlike previous studies, this work systematically compares both methods under controlled conditions to evaluate their efficiency, fiber integrity, and environmental impact. The extracted CNFs were characterized using X-ray diffraction (XRD), Fourier transform infrared spectroscopy (FTIR), scanning electron microscopy (SEM), energy-dispersive X-ray spectroscopy (EDX), dynamic light scattering (DLS), and zeta potential analysis. The FTIR spectra confirmed the presence of C–O–C fundamental vibrational stretching of cellulose and effective removal of non-cellulosic components such as lignin and hemicellulose. XRD results displayed the moderate crystalline nature of the extracted cellulose, with variation in intensity attributed to extraction technique and biomass type. Zeta potential analysis showed that CNFs extracted from eucalyptus bark via Soxhlet extraction exhibited superior colloidal stability (−32.5 mV), while those from sugarcane bagasse through chemo-mechanical treatment showed lower stability (−15.3 mV). These findings offer new insights into the method-material interaction and highlight the Soxhlet extraction route as more effective in producing stable, high-purity nanofibers. The protocols can be vital in reducing production costs and chemical utilization, enhancing material performance, and enabling large-scale application in packaging and biomedical industries.

## 1. Introduction

Advancements in the field of nanotechnology have developed global interest in the production of sustainable, eco-friendly, and biodegradable materials, driven by rising awareness of environmental conservation [1–4]. Therefore, the adoption of sustainable practices, resource optimization, and the introduction of green technology are outcomes of that global awareness [5–9]. The primary sources of bio-based materials are plant biomasses, which are mainly obtained from agricultural and non-agricultural wastes [10,11]. A significant portion of the generated residues/side-streams is not handled practically, and therefore contributes to ongoing environmental challenges [12–14]. One high-value application of these side-streams is the production of CNFs. CNFs are biodegradable, high-strength nanomaterials used in filtration membranes, packaging, composites, and biomedical devices [15–20].

Current production processes for CNFs include acid hydrolysis, microbial synthesis, and mechanical separation, but all approaches have certain limitations [21]. Acid hydrolysis poses environmental concerns by producing a significant amount of pollutants, while TEMPO oxidation, another chemical treatment process, is costly and provides less efficient recovery of nanofibers [22]. TEMPO oxidation specifically targets the primary -OH groups of cellulose using a stable nitroxyl radical catalyst, which generates large volumes of wastewater and is less suitable for large-scale production. Recent studies have emphasized that although TEMPO oxidation enhances fibrillation and introduces abundant carboxyl groups, improving CNF dispersibility, the process still requires optimization to reduce chemical consumption and effluent generation. Moreover, alternative eco-friendly oxidation routes are being explored to maintain similar surface functionality with lower environmental impact [23–26]. Similarly, microorganisms can also be used for the production of CNFs, but their handling can be hazardous, costly, and requires specialized equipment [20]. Microbial synthesis typically involves bacterial strains that produce extracellular cellulose, but the process is slow, highly sensitive to contamination, and difficult to control under industrial conditions [27]. These drawbacks make both techniques mentioned less effective and commercially unsuitable. Even the typical mechanical method requires high energy inputs and can compromise the cellular integrity of fibers [28]. Therefore, the optimization of mechanical techniques, due to huge industrial demand, for the production of CNFs becomes vital [29]. To reduce energy consumption and negative environmental impact, we have optimized protocols by integrating chemical and mechanical methods, and we aim to maintain environmental sustainability and feasibility.

In this study, two sustainable sources for CNF production were explored, including sugarcane bagasse (SCB) and eucalyptus bark (EUB). Globally, 540 million tons of dried sugarcane are produced annually, generating 280 kg of bagasse per ton [30]. Bagasse comprises cellulose, which accounts for 43.6% of its fiber content [31]. Eucalyptus is cultivated across 95 countries covering over 22.57 million hectares, which generates considerable bark waste from its widespread use in the timber and pulp industries [32,33]. However, these industries produce a significant proportion of waste that can be repurposed through valorization of agro-waste, and this process can contribute to sustainability by enhancing waste utilization and reducing environmental impact.

By valorizing agro-wastes such as SCB and EUB for the production of CNFs, the methods proposed in this study align with the principles of a circular bioeconomy. We introduce an optimized, low-energy extraction workflow that sequentially follows drying, milling, ethanol extraction, alkaline treatment, bleaching, and sonication. While the individual reagents such as NaOH, KOH, HNO₃, and NaClO₂ are well-documented in previous studies, our novelty lies in the systematic comparison of two extraction routes applied to two distinct biomass sources under unified and optimized conditions. This design enables a clearer understanding of how each protocol influences nanofiber quality, purity, and colloidal behavior. Moreover, unlike conventional methods, our approach minimizes chemical loading and avoids harmful oxidants such as TEMPO, providing a greener, more scalable process. The process is designed to reduce cost, valorize agro-waste, while enhancing yield and reproducibility. The main objective of this research is to comparatively assess chemo-mechanical and Soxhlet extraction strategies across SCB and EUB, and to evaluate their effect on CNF yield, characteristics, and stability. This comparative framework not only reveals process–property relationships but also offers a simplified platform for sustainable nanofiber production with improved industrial relevance.

## 2. Materials and methods

### 2.1. Materials

#### 2.1.1. Chemicals and reagents.

Sodium hydroxide (NaOH, ≥ 98%, 39.997 g/mol), nitric acid (HNO_3_, 65%, 63.01 g/mol), sodium nitrate (NaNO_3_, ≥ 99%, 84.99 g/mol), and absolute ethanol (C_2_H_5_OH, ≥ 99.8%, 46.07 g/mol) were acquired from VWR International (USA), while benzene (C_6_H_6_, ≥ 99.7%, 78.11 g/mol), hydrochloric acid (HCl, 35%, 36.46 g/mol), potassium hydroxide (KOH, ≥ 85%, 56.11 g/mol), glacial acetic acid (CH_3_COOH, ≥ 99%, 60.05 g/mol), and sodium chlorite (NaClO_2_, ≥ 80%, 90.44 g/mol) were purchased from Sigma Aldrich. All chemicals and reagents were of analytical grade and used without further purification. Distilled water was used throughout the reactions to avoid contamination.

#### 2.1.2. Plant biomass.

SCB was obtained from the local sugarcane company, Fecto Sugar Mills Ltd., Islamabad, Pakistan, while EUB was collected from a wood processing company, Woodsol (PVT) Ltd., Islamabad, Pakistan. Both plant biomasses were washed thoroughly with 70% ethanol before use in CNF preparation.

### 2.2. Methodologies

**Fig 1:**
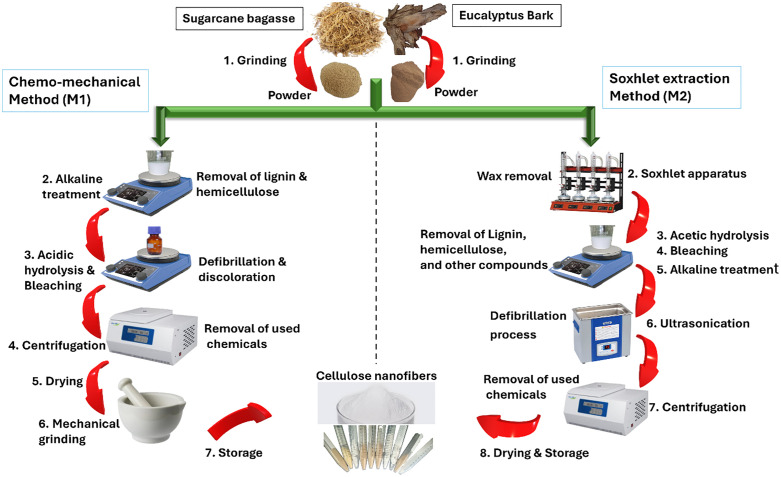
Schematic representation of the synthesis approaches for CNF production using EUB and SCB as raw materials.

CNFs were synthesized from two bio-based sources: EUB and SCB, using two distinct methods. One method involves a chemo-mechanical approach (M1) while the other employs a soxhlet extraction technique (M2) as presented in Fig 1. These two methodologies were optimized by protocols outlined by Kumar et al., 2020 and Chen et al., 2011, to improve the cost-effectiveness and energy efficiency of producing CNFs from both soft and hardwood [34,35].

#### 2.2.1. CNF production via chemo-mechanical method (M1).

The EUB and SCB were both cleaned with 70% ethanol and air-dried. They were then processed into a fine powder with a pulse grinder (SilverCrest® SC-150). The powdered samples of EUB and SCB (1 g each) underwent alkaline treatment with 2% NaOH and were later stirred for 24 h at room temperature. This step was aimed at removing the lignin and hemicellulose. After treating the sample with NaOH, it was rinsed and washed thoroughly with distilled water until the pH was carefully brought from basic to neutral. This neutralization step ensured the maximum removal of residual NaOH, which could have otherwise interfered with subsequent processing steps and compromised the quality of the CNFs. To produce CNFs from the treated samples, 1% v/v nitric acid (HNO_3_) was added for acidic hydrolysis gradually, and the sample was fully soaked in the acid. HNO_3_ was added to oxidize the cellulose, remove residual impurities, facilitate fiber separation into nanoscale dimensions, and enhance the dispersibility of the resulting CNFs. Then, 0.5% w/v NaNO_3_ was further added to facilitate oxidation induced by HNO_3_. This combination facilitated the in-situ formation of nitronium ions (NO₂⁺), which promoted oxidative cleavage of cellulose chains and aided in nanofiber formation. The reaction was carried out at 70 °C for 10 h under constant stirring in a strongly acidic environment to ensure effective delignification and controlled defibrillation without damaging the cellulose backbone. [36]. During this reaction, reddish-brown fumes appeared, which confirmed the production of nitrogen oxides during strong oxidative conditions. To prevent the removal of harmful gases, we sealed the flask with a glass stopper. After 10 h, the reaction was stopped by slowly adding distilled water to the flask. Following acidic treatments, samples were washed with ethanol-water (2:1 ratio) and centrifuged. Centrifugation at 9000 rpm (150 s^-1^) for 15 min was employed to separate the CNFs from the washing solvent and impurities. After drying at 50 °C for 2 h, they were ground by mortar and pestle and stored at −4 °C for further analysis. All the steps are presented in Fig 1.

#### 2.2.2. CNF production via soxhlet extraction method (M2).

The prepared powder samples of SCB and EUB were initially washed with ethanol and deionized water to remove dirt. Each sample (20 g) was gently heated at 80 °C in a sealed vessel for 6 h with a 2:1 mixture of benzene (C_6_H_6_) and ethanol (C_2_H_5_OH) to remove wax. The sealed vessel was used to avoid evaporation of ethanol at 80 °C. Benzene was selected in this protocol for its efficiency in wax removal, based on previous studies [37,38]. However, its use was strictly limited to a closed system, and the primary aim of our study was to compare extraction methods under controlled conditions. We acknowledge its toxicity and recommend replacing it with greener solvents like ethanol, limonene, ethyl acetate, or toluene in future scale-up applications to enhance sustainability. Following the initial steps, the lignin extraction was carried out by dissolving the samples in a 1% w/v sodium chlorite (NaClO_2_) solution, supplemented with 10% v/v acetic acid (CH_3_COOH), at 60 °C for 1 h with continuous stirring. The mixture was then washed with distilled water and filtered, separating the residue from the washing solvent using filter paper. The residue underwent a bleaching process two times using the same procedure to ensure thorough discoloration. The bleaching treatment effectively decomposed phenolic compounds or molecules containing chromophoric groups in the lignin, while also eliminating the byproducts of this decomposition, resulting in the whitening of the pulp. Thereafter, the samples were treated with 3% sodium hydroxide (NaOH) solution at 80 °C for 2 h, followed by 6% potassium hydroxide (KOH) at the same temperature for another 2 h to remove hemicellulose, starch, and pectin. Following the KOH treatment, impurities were removed through filtration with deionized water, followed by continuous washing to neutralize the pH. The CNFs were sonicated in distilled water at 60 °C for 30 min to reduce the cellulose fibers to the nanoscale level. After sonication, the samples were centrifuged at 13000 rpm (216.7 s^-1^) for 20 min to eliminate water and other chemicals. Subsequently, the samples were washed, dried, ground by mortar and pestle, and finally stored at −4 °C for further analysis. All the steps are presented in Fig 1.

#### 2.2.3. Characterization of the prepared CNFs.

**X-ray diffraction (XRD)**: The diffractogram was recorded using an XRD spectroscope (Bruker D8 ADVANCE, Germany). The samples were scanned over 2θ ranges from 10° to 40°. Gaussian-shaped peaks were fitted after deconvolution using OriginPro software.

**Fourier transform infrared (FTIR) spectroscopy**: FT-IR spectra of CNFs obtained from SCB and EUB samples were recorded using an FTIR Spectrophotometer Nicolet iS5N, (Thermo Scientific). Samples were ground with KBr, compacted into discs, and analyzed. Wavelengths of spectra were recorded (4000–500 cm^−1^) using OriginPro software.

**Scanning electron microscopy (SEM)**: CNFs obtained from SCB and EUB samples were assessed through a NOVA NANO SEM 230 microscope. Before examination, a fine gold coating was applied to the samples using an ion sputter coater at a low deposition rate to enhance conductivity.

**Energy Dispersive X-ray Spectroscopy (EDX)**: The EDX analysis was performed to obtain information about the elemental composition of CNFs, prepared by different methods using two different sources, using NOVA NANO SEM 230 instrument.

**Hydrodynamic size and zeta potential**: Samples underwent ultrasonic dispersion for 15 min, followed by transfer into a transparent quartz tube. Submicron particle size and zeta potential analyzer (Malvern Instruments Ltd., Mastersizer 2000d model APA2000, UK) was used to measure particle size distribution. The solution, prepared as a 0.5 wt % CNF solution with pH adjusted to 7–8, was tested for zeta potential. A small amount of the solution was introduced into the measurement device via a syringe. The instrument employs photon correlation spectroscopy and electrophoretic light scattering to determine both particle size and zeta potential directly from the suspension without requiring dilution.

## 2.3. Ethical approval

This study did not involve any experiments on humans or animals; therefore, ethical approval was not required.

## 3. Results and discussion

### 3.1. Synthesis of CNFs via chemo-mechanical and soxhlet extraction methods

**Fig 2:**
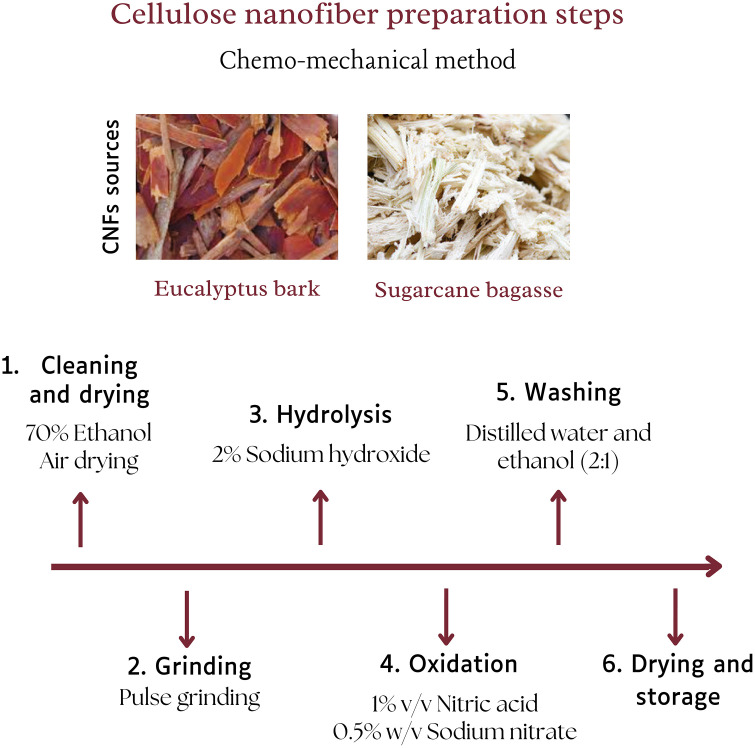
Steps involved in the synthesis of CNFs by chemo-mechanical treatment.

**Fig 3:**
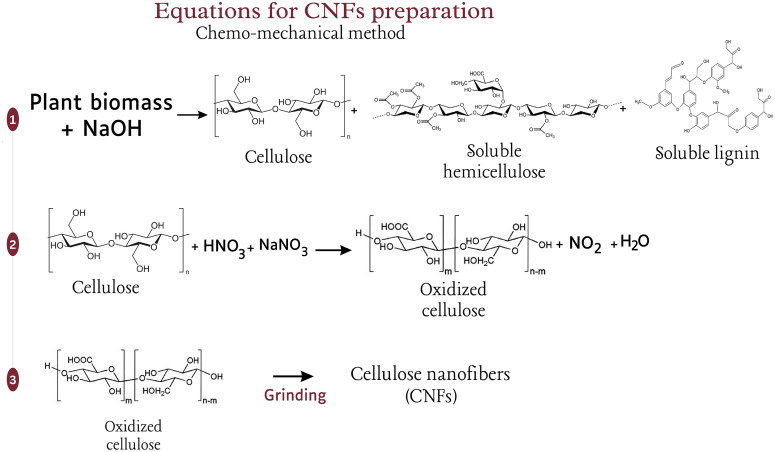
Possible equations leading to the synthesis of CNFs by chemo-mechanical treatment.

The preparation of CNFs from EUB and SCB was performed by employing two different techniques, which include chemo-mechanical treatment and soxhlet extraction. Both mentioned techniques were employed as reliable and cost-effective approaches to produce CNFs, as each approach offers unique advantages that signify their importance. In the chemo-mechanical method, the first step involves the cleaning and drying of the raw materials, followed by grinding and later hydrolysis using 2% NaOH to remove lignin and hemicellulose. Hydrolysis is crucial for breaking down the complex structure of the raw materials and facilitating the release of cellulose fibers. Subsequently, the oxidation of cellulose by 1% v/v nitric acid and 0.5% w/v sodium nitrate at 70 °C facilitated the formation of nanoscale cellulose. Later, neutralization and proper washing ensured the effective removal of residual chemicals while maintaining the structural integrity of cellulose. The prepared CNFs were dried and ground to a fine powder by mortar and pestle, and later stored at −4 °C. Steps are comprehensively presented in Fig 2, and possible equations for CNFs production are presented in Fig 3. The minimal use of chemical solvents and energy makes this chemo-mechanical technique cost-effective and an environmentally friendly option.

**Fig 4:**
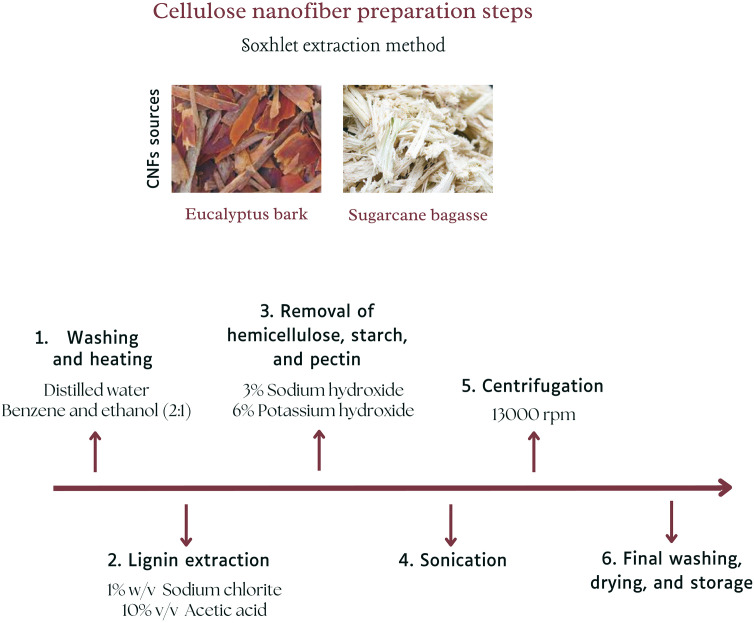
Steps involved in the synthesis of CNFs by the Soxhlet extraction method.

**Fig 5:**
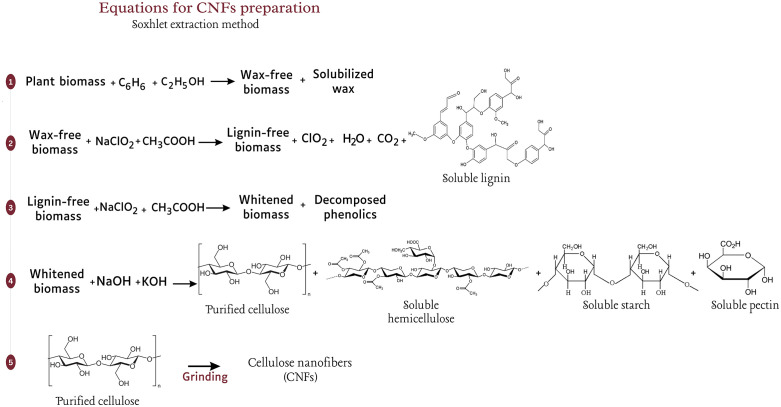
Possible equations leading to the synthesis of CNFs by the Soxhlet extraction method.

In contrast, the Soxhlet extraction method (M2) began with washing the powdered samples with ethanol and deionized water, followed by heating with a benzene-ethanol mixture to remove waxes. This thorough cleaning was essential for preparing the raw materials for subsequent treatments. Lignin extraction using a sodium chlorite solution with acetic acid at 60 °C effectively removed non-cellulosic components, which was critical for obtaining pure cellulose. Multiple bleaching cycles further ensured the removal of chromophoric groups, resulting in visually pure and bright CNFs. Sequential treatment with sodium hydroxide and potassium hydroxide removed hemicellulose, starch, and pectin, leaving behind a high-quality cellulose matrix. The final steps of sonication to reduce the cellulose fibers to the nanoscale, centrifugation to remove water and residual chemicals, followed by drying and storage, were vital for achieving the desired nanoscale dimensions and maintaining the purity and quality of the CNFs. All the steps are carefully presented in Fig 4, and possible equations for CNF synthesis are presented in Fig 5. The Soxhlet extraction method was important for its comprehensive approach to removing non-cellulosic components, ensuring the production of high-purity CNFs suitable for advanced applications.

Both methods were meticulously designed to remove non-cellulosic components and achieve high-quality CNFs. M1 focused on a combination of chemical and mechanical treatments for efficiency, while M2 employed a more solvent-intensive approach for thorough lignin and hemicellulose removal. The importance of these methods lay in their ability to tailor the extraction process to the specific needs of CNFs production, ensuring that the final product was of high quality and suitable for various industrial applications.

### 3.2. Comparative analysis with reported CNFs extraction methods

Various extraction approaches are documented in the current literature, each associated with certain pros and cons are presented in Table 1 and Table 2.

**Table 1:** CNFs extraction from TEMPO and microwave-assisted methods.

Study	Method	Biomass	Chemicals	Temp/ time	Yield
[39]	TEMPO	*Paulownia fortunei*	TEMPO, NaBr, NaClO, NaOH, Na_2_SO_3_, H_2_O_2_	90°C/ 5 h	40%
[40]	TEMPO	*Corchorus olitorius*	TEMPO, NaBr, NaClO, NaOH	Room temperature/ 24 h	60-65%
[41]	TEMPO	*Eucalyptus globulus*	TEMPO, NaBr, NaClO, NaOH, HCl, KI, Na_2_SO_3_	25°C/ 24 h	N/A
[42]	Microwave-assisted	*Eucalyptus sp*	toluene, H_2_SO_4_, para-aminobenzoic acid	−110°C/ 72 h	73.3%
[43]	Microwave-assisted with TEMPO	Potatoe	TEMPO, NaBr, NaOH, H_2_O_2_	N/A	84.44%

**Table 2:** CNFs extraction from enzymatic, electrospinning, and carbon dioxide methods.

Study	Method	Biomass	Chemicals	Temp/ time	Yield
[44]	Enzymatic approach (cellulase, xylanase)	sugar beet pulp	m-hydroxybiphenyl, NaClO_2_, H_2_SO_4_	85°C/ 2 h	61.4%
[31]	Enzymatic approach (xylanase, cellulase)	*Salicornia ramosissima*	K_3_PO_4_, HCl, (NH₄)₂SO₄, NaOH, H_2_O_2_	100°C/ 1 h	5.1%
[45]	Electrospinning	*Agave tequilana*	1-butyl-3-methylimidazolium chloride, trifluoracetic acid, NaOH, NaClO, CH_3_COOH	60°C/ 24 h	N/A
[46]	Carbon dioxide approach	Natural fiber carpet wastes	NaOH, NaClO_2_, H_2_O_2_, CH_3_COOH	60°C/ 2 h	92%

The yield of CNFs varied depending on the extraction method and the chemical system used. In the present study, Soxhlet-based samples CNF-E(M2) and CNF-S(M2) showed yields of 33.6% and 30.2%, while chemo-mechanical samples CNF-E(M1) and CNF-S(M1) gave slightly lower yields of 27.4% and 24.8%, respectively. Compared to literature-reported yields from TEMPO-based methods (40–65%) and microwave-assisted extractions (up to 84.4%), our values are moderate but achieved under milder, greener conditions. Similarly, enzymatic and CO₂-based methods have reported highly variable yields ranging from 5.1% to 92%, often depending on the biomass type and reaction severity. These differences highlight how intensive chemical treatments or prolonged reaction times may boost yield but also raise sustainability concerns. In our case, the absence of strong oxidizers like NaClO and the use of lower-temperature alkaline steps may have limited cellulose breakdown, resulting in slightly reduced yields but with fewer chemical residues and better process safety.

### 3.3. Optical, physicochemical, and morphological characterization of CNFs

#### 3.3.1. XRD analysis.

**Fig 6:**
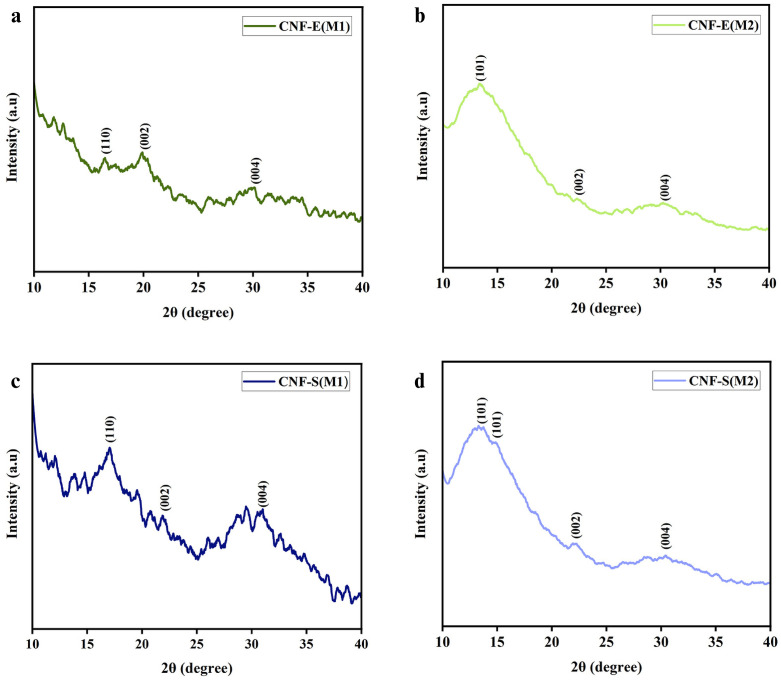
XRD spectra of EUB (CNF-E) and SCB (CNF-S) sourced CNFs using chemo-mechanical treatment (M1) and Soxhlet extraction (M2). **(a)** CNF-E(M1), **(b)** CNF-E(M2), **(c)** CNF-S(M1), and **(d)** CNF-S(M2).

CNFs obtained from extracts of EUB (CNF-E) and SCB (CNF-S) by chemo-mechanical (M1) and Soxhlet extraction (M2) were assessed, to analyze the intensity of diffraction peaks, by X-ray diffractometer as presented in Fig 6. Samples with similar sources showed spectra with slightly different peak intensities, which may happen due to the induction of different preparation methodologies. For instance, CNF-E(M1) displayed peaks at 2θ values of 16.48°, 20°, and 30.01° corresponding to (110), (002), and (004) planes while CNF-E(M2) presented peaks at 2θ values of 13.3°, 22.2°, and 30.46° corresponding to (101), (002), and (004) planes, respectively [47,48]. Similar peaks were documented in other studies where CNFs were originally sourced from EUB, which confirms the homogeneity of our research [47,49]. Similarly, CNF-S(M1) displayed visible peaks at 2θ values of 16.97°, 22.21°, and 31.01° corresponding to (110), (002), and (004) planes respectively while CNF-S(M2) presented diffraction peaks at 2θ values of 13.49°, 14.85°, 22.19°, and 30.40° corresponding to (101), (101), (002), and (004) [50,51]. Similar peaks were documented in other studies where CNFs were originally sourced from SCB, which confirms the homogeneity of our research [50,52]. The noise in XRD spectra of CNFs prepared via the chemo-mechanical method may arise due to the residual non-cellulosic impurities such as lignin and hemicellulose. These impurities can lead to disordered scattering while increasing noise in the spectra. The presence of noise in XRD spectra is consistent with previous studies reporting that the presence of residues such as lignin and hemicellulose can reduce the crystallinity and affect the structural integrity of CNFs [53,54]. The XRD data thus confirms that the Soxhlet extraction technique produced comparatively pure CNFs with fewer impurities, while the chemo-mechanical method was less feasible.

#### 3.3.2. FTIR analysis.

**Fig 7:**
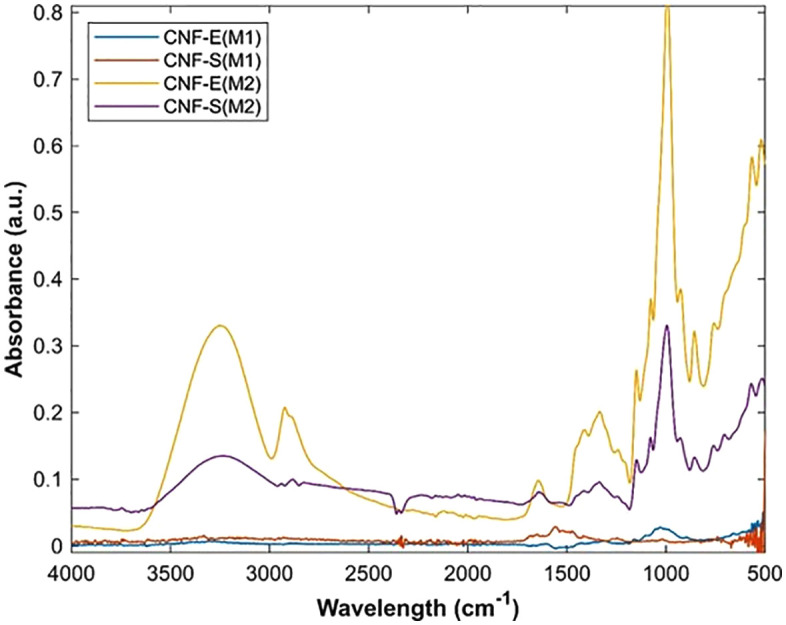
FTIR spectra of EUB (CNF-E) and SCB (CNF-S) sourced CNFs using chemo-mechanical treatment (M1) and Soxhlet extraction (M2).

CNFs obtained from extracts of EUB (CNF-E) and SCB (CNF-S) by chemo-mechanical (M1) and Soxhlet extraction (M2) were subjected to functional group identification through FTIR spectroscopy as presented in Fig 7. For M2 samples CNF-E(M2) and CNF-S(M2), the spectra show sharp and well-defined peaks related to cellulose. In CNF-E(M2), strong C–O–C ether stretching peaks were observed at 776 cm ⁻ ¹ and 1031 cm ⁻ ¹, which confirm the β-(1 → 4) glycosidic linkage of cellulose [55]. Bands around 1312–1430 cm ⁻ ¹ and 1602 cm ⁻ ¹ correspond to –CH₃, CH₂–OH, and C = O bonds, while broad –OH stretching in the range of 3200–3500 cm ⁻ ¹ indicates hydrogen bonding, which is typical of cellulose with good purity [47,56]. CNF-S(M2) also showed intense C–O–C stretching at 830 cm ⁻ ¹ and 1019 cm ⁻ ¹, along with –CH₂ and C = O peaks at 1351 cm ⁻ ¹ and 1617 cm ⁻ ¹. The broad –OH band confirms the presence of strong hydrogen bonding, which is usually linked to higher cellulose crystallinity [56–59]. These sharp and distinct peaks suggest that Soxhlet extraction helped in better removal of lignin and hemicellulose, leaving cellulose-rich nanofibers, as also supported in earlier reports.

In contrast, the spectra of M1 samples CNF-E(M1) and CNF-S(M1) showed only weak and unclear peaks, with very low absorbance across the fingerprint region. Faint signals near 898–1018 cm ⁻ ¹ (C–O–C stretching) and around 1411–1473 cm ⁻ ¹ (CH₂–OH bending) were visible [57], but the intensity was not strong enough to clearly highlight cellulose features. This can be explained by the incomplete removal of lignin and hemicellulose during the chemo-mechanical process, which reduces cellulose purity and causes overlapping of signals. The weaker absorbance also suggests that the concentration of exposed cellulose nanofibers was lower in these samples compared to M2.

Overall, the FTIR data show that the M2 method (Soxhlet extraction) produced CNFs with much clearer cellulose peaks, while the M1 method gave only minor peaks due to remaining amorphous components.

#### 3.3.3. SEM analysis.

**Fig 8:**
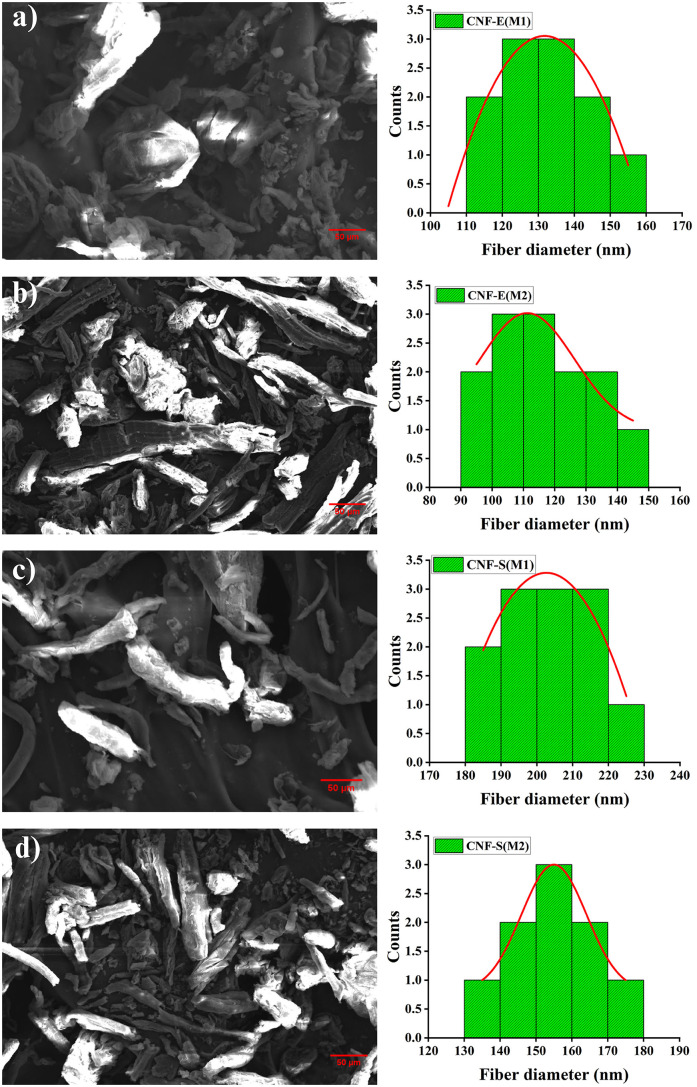
SEM micrographs and histograms of (a) CNF-E(M1), (b) CNF-E(M2), (c) CNF-S(M1), and (d) CNF-S(M2).

The morphology of CNFs, synthesized by chemo-mechanical treatment (M1) and Soxhlet extraction (M2) methods was analyzed by SEM as presented in Fig 8. The SEM micrographs of CNFs obtained by the Soxhlet extraction method displayed needle-like, well-defined morphology, and this can be attributed to the efficient removal of various non-cellulosic components such as lignin and hemicellulose; however, CNFs obtained by the chemo-mechanical method showed less pure needle-like fibers, which indicate the presence of impurities such as lignin and hemicellulose, as confirmed by FTIR and XRD spectra. Similarly, defibrillation of EUB and SCB fibers by ultrasonication led to the synthesis of finer and uniform fibrils. Such structural morphology is usually associated with improved mechanical strength and stability of composites and sustainable packaging materials. These findings are consistent with Pessan et al., 2023, who reported similar morphology in oxidized CNFs that were derived from SCB [59]. This result indicates that high purity of CNFs can be obtained by efficient and cost-effective approaches such as chemo-mechanical and Soxhlet extraction methods, which are used in the study.

#### 3.3.4. EDX analysis.

**Fig 9:**
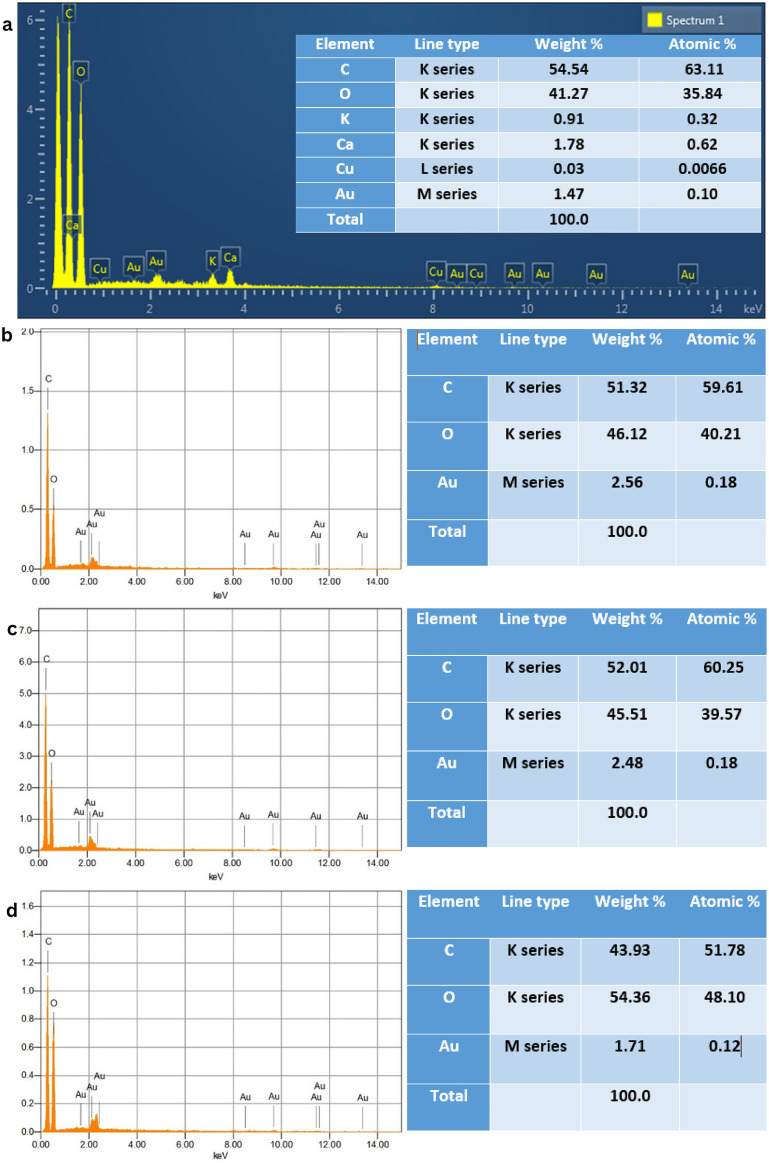
EDX spectra of (a) CNF-E(M2), (b) CNF-E(M1), (c) CNF-S(M2), and (d) CNF-S(M1).

The elemental composition of CNFs was confirmed by using EDX spectroscopy, as presented in Fig 9. For CNF-E(M2), C, O, K, Ca, Cu, and Au elements were observed with weight% of 54.54, 41.27, 0.91, 1.78, 0.03, and 1.47%, respectively. The presence of a small amount of K in the EDX of CNF-E(M2) may be attributed to the use of KOH during the synthesis process, while the trace amount of Cu may be attributed to the Cu-sample holder used in EDX analysis, and Ca might come from impurities in the raw material. Similarly, Au is detected due to sputter coating on nanofiber samples for better conduction. Subsequently, the EDX spectra of CNF-E(M1) showed the presence of C, O, and Au elements with weight% of 51.32, 46.12, and 2.56%, respectively. Similarly, in CNF-S(M2), C, O, and Au elements were observed with weight% of 52.01, 45.51, and 2.48% respectively, while EDX spectra of CNF-S(M1) showed the presence of C, O, and Au elements with weight% of 43.92, 54.36, and 1.71% respectively. EDX spectra prominently showed C and O, which are major constituents of CNFs, thus confirming the synthesis of CNFs with minor impurities.

#### 3.3.5. Hydrodynamic size and zeta potential analysis.

The CNFs obtained by chemo-mechanical and Soxhlet extraction methods were subjected to hydrodynamic size and zeta potential analysis by the dynamic light scattering (DLS) method. As documented in Table 3, CNF-E(M1) and CNF-E(M2) showed an average size of 132.5 nm and 88.48 nm, while CNF-S(M1) and CNF-S(M2) showed an increased size of 258.1 nm and 151.89 nm, respectively. The documented results are consistent with previous literature by Tibolla et al., 2014 who reported similar hydrodynamic size ranges for CNFs sourced from banana peels [60]. Interestingly, CNFs obtained by different extraction methods displayed negatively charged density, as presented in Table 3. For instance, CNF-E(M1) and CNF-E(M2) showed zeta potential of −31.4 mV and −32.5 mV, whereas CNF-S(M2) showed a slightly lower zeta potential of −29.3 mV. CNF-S(M1) displayed a comparatively lower zeta potential of −15.3 mV, which indicates a greater tendency for aggregate formation. Zeta potential (ζ) shows the electric potential among the stationary charged layers of nanofibers, and it plays a significant role in defining the dispersion and interaction capacity of nanofibers with the surrounding environment [7]. Our findings thus conclude that variations in size and zeta potential of different CNFs may be attributed to the extraction methods, biomass sources, and residual non-cellulosic components.

**Table 3:** Hydrodynamic size and zeta potential of different CNFs.

Samples	Hydrodynamic size (nm)	Zeta potential (mV)
CNF-E(M1)	132.5	−31.4
CNF-E(M2)	88.48	−32.5
CNF-S(M1)	259.1	−15.3
CNF-S(M2)	151.89	−29.3

## 4. Limitations and future perspective

While this study offers valuable insights into sustainable CNFs production from agro-waste using chemo-mechanical and Soxhlet extraction methods, a few limitations point the way for future improvements. Due to limited access to high-end instrumentation, we were unable to perform additional transmission electron microscopy, however, our characterizations provided clear evidence of successful CNFs synthesis. Mild noise observed in the XRD spectra is consistent with reported literature and likely due to residual non-cellulosic content, suggesting room for further purification to enhance crystallinity. The use of benzene in Soxhlet extraction, though confined to a closed system, raises safety concerns, and future research should explore greener alternatives, like toluene or limonene. Additionally, verification of solvent removal through techniques like GC-MS was not feasible in this study but is recommended for future work to strengthen environmental assurances. Overall, despite these constraints, the study successfully demonstrates optimized protocols that can guide greener, scalable, and more efficient CNFs extraction in the future.

## 5. Conclusion

In this study, we successfully extracted CNFs from EUB and SCB using two distinct methodologies, including the chemo-mechanical treatment and Soxhlet extraction. A thorough comparative assessment revealed that the Soxhlet method was more effective in removing non-cellulosic components, yielding nanofibers with greater purity and structural clarity. In contrast, the chemo-mechanical approach, while simpler, resulted in nanofibers with residual impurities, as evidenced by FTIR and the increased noise observed in XRD spectra. These findings highlight the importance of method selection in determining nanofiber quality and colloidal behavior. Overall, the study provides a meaningful contribution by demonstrating optimized, low-energy pathways for valorizing agricultural residues into functional nanomaterials, supporting future efforts in sustainable material development and scalable nanofiber production.
